# Minor Phytocannabinoids: A Misleading Name but a Promising Opportunity for Biomedical Research

**DOI:** 10.3390/biom12081084

**Published:** 2022-08-06

**Authors:** Diego Caprioglio, Hawraz Ibrahim M. Amin, Orazio Taglialatela-Scafati, Eduardo Muñoz, Giovanni Appendino

**Affiliations:** 1 Dipartimento di Scienze del Farmaco, Università del Piemonte Orientale, Largo Donegani 2, 28100 Novara, Italy; 2 Dipartimento di Farmacia, Università di Napoli Federico II, Via Montesano 49, 80131 Napoli, Italy; 3 Instituto Maimónides de Investigación Biomédica de Córdoba, 14004 Córdoba, Spain; 4 Departamento de Biología Celular, Fisiología e Inmunología, Universidad de Córdoba, 14071 Córdoba, Spain; 5 Hospital Universitario Reina Sofía, 14004 Córdoba, Spain

**Keywords:** phytocannabinoids, minor cannabinoids, precannabinoids, decarboxylation, cannabinoid receptors, thermo-TRPs, PPARγ

## Abstract

Despite the very large number of phytocannabinoids isolated from Cannabis (*Cannabis sativa* L.), bioactivity studies have long remained focused on the so called “Big Four” [Δ^9^-THC (**1**), CBD (**2**), CBG (**3**) and CBC (**4**)] because of their earlier characterization and relatively easy availability via isolation and/or synthesis. Bioactivity information on the chemical space associated with the remaining part of the cannabinome, a set of ca 150 compounds traditionally referred to as “minor phytocannabinoids”, is scarce and patchy, yet promising in terms of pharmacological potential. According to their advancement stage, we sorted the bioactivity data available on these compounds, better referred to as the “dark cannabinome”, into categories: discovery (in vitro phenotypical and biochemical assays), preclinical (animal models), and clinical. Strategies to overcome the availability issues associated with minor phytocannabinoids are discussed, as well as the still unmet challenges facing their development as mainstream drugs.

## 1. Introduction

**Figure 1:**
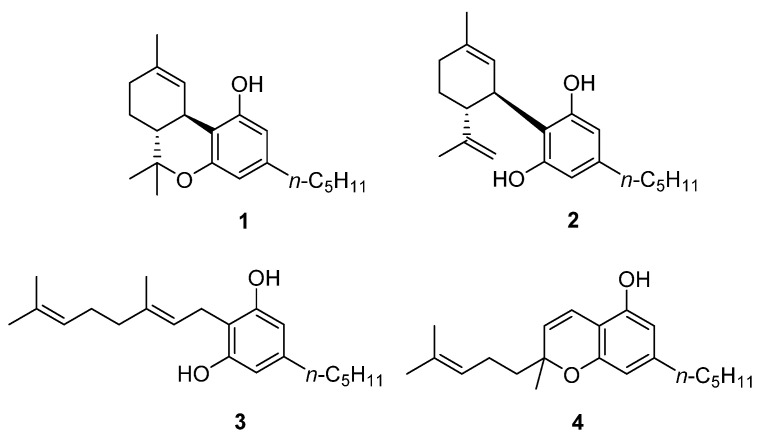
Structures of the “Big Four” phytocannabinoids: Δ^9^-THC (**1**), CBD (**2**), CBG (**3**), and CBC (**4**).

Early research into phytocannabinoids focused almost exclusively on Δ^9^-tetrahydrocannabinol (Δ^9^-THC, **1**, Figure 1), the narcotic principle of marijuana [1], branching out to related compounds from cannabis (cannabidiol (CBD, **2**, Figure 1) cannabigerol (CBG, **3**, Figure 1) and cannabichromene (CBC, **4**, Figure 1)) only sporadically because of their lack of narcotic properties, which was the major biological end-point associated with cannabis constituents for too long [1]. Progress in our understanding of the biological mechanism(s) underlying the narcotic properties of Δ^9^-THC led to the discovery of the endocannabinoid system, whose complexity, homeostatic role, and potential for drug discovery [2] spurred a re-evaluation of the other three major cannabinoids from cannabis. These studies eventually led to the mainstream pharmaceutical development of CBD (**2**) as a standardized extract (Sativex) and as a stand-alone active pharmaceutical ingredient (API) (Epidiolex) [3], as well as to a growing body of literature on CBG (**3**) and CBC (**4**). Sativex, a combination of Δ^9^-THC- and CBD-standardized cannabis extracts, is used to treat spasticity associated with multiple sclerosis, while Epidiolex is the drug of choice for the management of some rare genetic forms of epilepsy [3].

In the wake of the successful development of CBD (**2**) as a mainstream drug, cannabis research is now witnessing a novel wave of interest, namely the wave on “minor phytocannabinoids”. Despite the wealth of recent articles on these compounds, a precise definition of “minor phytocannabinoid” is still missing, while information on their chemistry and biological profile remains patchy. This prompted us to critically review the semantics of “minor phytocannabinoids” and to realistically evaluate not only the major findings in the area but also which gray areas remain to be clarified to fuel development.

The name “minor phytocannabinoids” or “rare cannabinoids” has been used to define phytocannabinoids different from the so called “Big Four”, namely Δ^9^-THC (**1**), CBD (**2**), CBG (**3**) and CBC (**4**) [4]. Since these compounds were structurally elucidated at the outset of the structure elucidation of phytocannabinoids (CBD in 1963, CBG and Δ^9^-THC in 1964 and CBC in 1966) [1], the assumption is that longevity and availability have combined into a better biological profile compared to more recently isolated phytocannabinoids [5]. As a result, the name “minor phytocannabinoids” has been used to indicate cannabinoids whose biological profile is poorly investigated; therefore, “minor” refers not to their actual concentration in cannabis, but *in the literature* (!). Several issues combine to make this definition misleading. To start with, CBC (**4**) is not a major constituent of cannabis. It was long confused with CBD (**2**) because the two compounds have the same molecular weight and very similar GC chromatographic retention time, but the actual concentration of CBC in cannabis is generally up to two orders of magnitude lower than the one of the dominant phytocannabinoid [6]. Furthermore, while it was possible to generate almost pure breeds of cannabis containing CBD (**2**), Δ^9^-THC (**1**) and CBG (**3**), this has proved much more difficult for CBC (**4**), whose production is associated with a juvenile trait orthogonal to the Mendelian allelic heritage associated with the production of CBD, Δ^9^-THC and CBG [7]. Therefore, the inclusion of CBC in the “Big Four” is basically only associated with its early isolation and easy availability via synthesis [6]. Next, the name “minor cannabinoids” seems out of place for acidic cannabinoids, the native form of phytocannabinoids, since the “Big Four” are derived from the decarboxylation of their corresponding acidic phytocannabinoids, whose concentration in the plant biomass is therefore at least equal to the one of their decarboxylated derivatives [5]. Furthermore, selective breeding can generate chemovarieties of cannabis accumulating cannabinoids normally only present in small amounts in native landraces of the plant, as shown for propyl phytocannabinoids (viridinoids, indicated with a V after the three letters acronym of the corresponding pentyl analogue, e.g., CBDV, **9a**) [8]. Finally, there is a disparity of information on Δ^9^-THC and CBD compared to CBG and CBC, and rather than “Big Four”, we should speak of “Big Two” [9]. However, since the name “minor cannabinoids” and its association to the “Big Four” is well established in the literature, a more rational alternative to define these compounds is probably bound to remain disattended, even though “dark cannabinome” would be a better alternative to refer to the vast area of the phytocannabinoid biological space still poorly explored in terms of bioactivity. This area of the chemical space encompasses ca. 150 phytocannabinoids isolated from various accessions of cannabis [5], but information on the biological profile of most of them is missing or is limited to a predictable lack of narcotic properties. To avoid duplication with existing reviews in the area [4,9,10,11], we sort the information available on minor phytocannabinoids according to the type of bioactivity documented for in vitro and in vivo studies. This gives readers a vivid picture of the potential of the “minor phytocannabinoids” pool for biomedical research, helping the identification of bioactivity hotspots worthy of further attention. In addition, since many “minor phytocannabinoids” are only available in trace via isolation from cannabis biomasses, alternative strategies to provide sufficient material to support additional studies will be critically discussed.

## 2. Discovery Studies

### 2.1. Biochemical Assays

The archetypal bioactivity transducers of phytocannabinoids are the cannabinoid receptors CB1 and CB2 [2], but interaction with additional GPCRs as well as ion channels, enzymes and transcription factors has been reported, contributing, to a various extent, to their in vivo biological profile [9]. This diversity of targets translates into a potential for the management of different diseases. Very broadly speaking, the direct activation of CB1 is associated with narcotic (euphoric) effects as well as analgesic, orexic and anxiety-modulating activity, with narcotic activity being absent in allosteric activators. Conversely, the activation of CB2 is related to immunomodulation and anti-inflammatory activity. The modulation of various thermo-TRPs is relevant for the management of pain and inflammation, while interaction with various PPARs is associated with anti-diabetic activity. The combination of these “primary” activities underlies the clinical potential of phytocannabinoids for complex conditions which lack a single therapeutic target, such as pathologies associated with neurodegeneration for compounds combining CB2 and PPARγ activation [12].

#### 2.1.1. Type-1 Cannabinoid Receptor (CB1)

**Figure 2:**
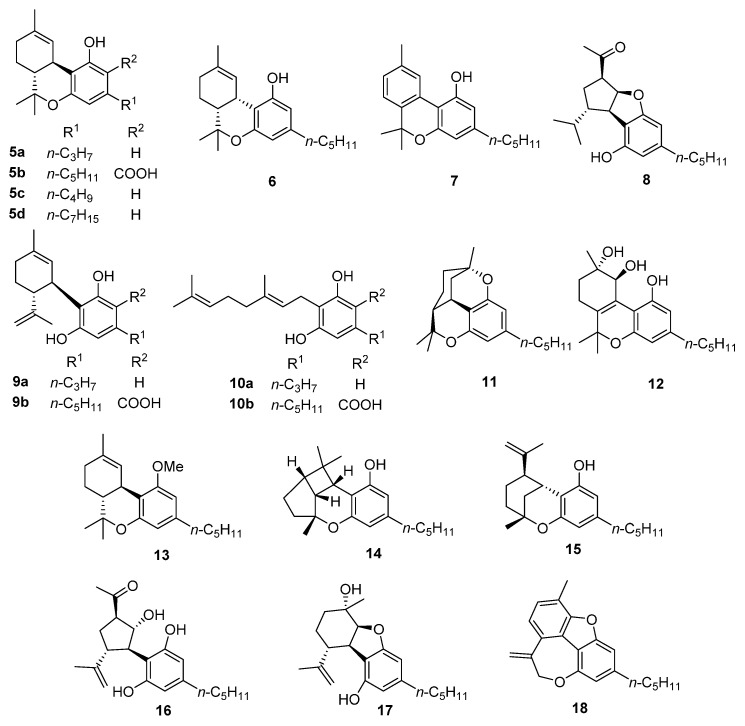
Minor phytocannabinoids cited in the text.

Higher and lower homologues of Δ^9^-THC activate CB1 with similar (Δ^9^-tetrahydrocannabibutol (nor-Δ^9^-THC) and Δ^9^-THCB, **5c**, Figure 2) [13] or even increased (Δ^9^-tetrahydrocannabiphorol (homo-Δ^9^-THC) and Δ^9^-THCP, **5d**, Figure 2) potency compared to the parent compound [14], but contrasting data have been reported on the CB1 activity of several minor phytocannabinoids. The discrepancy is seemingly related to differences in radioligand concentration, the expression of the receptor in the cell line used, the protocol of analysis, or to a combination of these factors. Additionally, phytocannabinoids can interact with both orthosteric and allosteric sites of cannabinoid receptors, complicating the interpretation of the experimental data, since the variety of binding modes translates into qualitatively different effects depending on the signaling pathway involved in the activation [15]. Additional concern regards the bias associated with activation, which was shown to depend on the receptor involved (CB1, CB2 or heterodimers CB1–CB2) as well as on the reference compound used to calculate the bias factor [15]. As a result, the molecular pharmacologist of some minor phytocannabinoids such as Δ^9^-THCV (**5a**) remains ambiguous. A clear and simple distinction should, in any case, be made between activity associated with the displacement of a radiolabeled ligand (e.g., CP 55,940) and activity associated with a functional biochemical assay (β-arrestin2 recruitment and cAMP accumulation). Thus, Δ^9^-THCA-A (**5b**) retains significant activity in displacement-based assays, while Δ^9^-THCA-A (**5b**) is even more powerful than Δ^9^-THC (**1**) in these assays, but both compounds are almost inactive in functional assays of β-arresting 2 recruitment, and additional studies identified Δ^9^-THCV (**5a**) as a CB1 antagonist or very weak agonist and Δ^9^-THCA-A (**5b**) as an allosteric activator of CB1 [15]. (−)-*cis*-Δ^9^-THC (**6**, Figure 2) duplicated, albeit with less potency, the activity of (*trans*)-Δ^9^-THC at CB1 [16], while CBN (7) is a weak partial agonist of CB1 in both radioligand-based and in functional assays [17]. Conversely, anhydrocannabimovone (8, Figure 2) shows significant binding activity at CB1 [18]. No significant marginal activity in CB1 assays has been reported for other minor phytocannabinoids, including CBGA (10b, Figure 2) and CBDV (9a, Figure 2) [17].

#### 2.1.2. Type 2-Cannabinoid Receptor (CB2)

Apart from Δ^9^-THC lower and higher homologues, which basically duplicate the CB1-CB2 activating ratio of the parent compound [13,14], most minor phytocannabinoids show higher affinity for CB2 compared to CB1 [17]. Significant activity in binding and functional assays has been reported for the acidic phytocannabinoids Δ^9^-THCA-A (**5b**) and CBDA (**9b**), with CBDV (**9a**) showing minor potency [17]. (−)-*cis*-Δ^9^-THC (**6**) [16], CBDV (**9a**), CBN (**7**) and anhydrocannabimovone (**8**) [18] showed significant activity in binding assays. Marginal activity was, conversely, reported for CBGA (**10b**), as well as for other minor phytocannabinoids [17].

#### 2.1.3. Non-CB GPCRs (GPR6, GPR18, GPR55 and GPR119)

Phytocannabinoids bind with different affinity to various CPCRs in addition to CB1 and CB2, and especially GPR55, sometimes even considered a third cannabinoid receptor [17]. GPR55 is expressed in the brain and is sensitive to phytocannabinoids (inhibition) and to lysophosphatidylinositol (LPI, activation), with activation triggering the mobilization of intracellular calcium. GPR55 acts as a “gate” to regulate calcium-dependent glutamate release, and GPR55 inhibition decreases CNS glutamate release and excitability, potentially translating into anti-epileptic activity [19]. CBDV (**9a**) showed activity similar to CBD and Δ^9^-THC toward GPR55 (antagonist) and GPR6 (inverse agonist), but the precise clinical translation of this activity is difficult to assess given the promiscuous nature of these compounds. Remarkably, the activation of the LPI/GPR55 pathway was shown to stimulate angiogenesis in in ovarian carcinoma, and its inhibition could be of oncological relevance [20]. The anti-inflammatory activity of THCA was inhibited by the GPR55 antagonist CID16020046, but not by the CB1 and CB2 receptor antagonist Rimonabant, suggesting a pre-eminent role of the so-called “third CB receptor” in this activity [20]. Of relevance is also the interaction of phytocannabinoids with GPR18, now renamed the NAGly receptor after its deorphanization with the endogenous lipid *N*-arachidonoylglycine, due to the important role of this receptor in the control of eye pressure [21]. GRP18 is activated by the non-natural cannabinoid abnormal-CBD as well as by the minor phytocannabinoid cannabicitran (**11**) [21].

#### 2.1.4. TRP Channels

These membrane proteins are involved in the transduction of multiple chemical and physical stimuli as well as in the generation of pain signals. In addition, their activation can trigger and sustain inflammation. Activation followed by desensitization is therefore important for chronic painful conditions such as neuropathic pain [22]. Several minor phytocannabinoids interact with a subset of TRPs involved in sensing temperature (thermo-TRPs, TRPV1-V4, TRPM8 and TRPA1). Most phytocannabinoids inhibit TRPM8 and activate, to a various extent, TRPV1-V4 and TRPA1, with potency being especially relevant for TRPV4 [22,23]. Within the “Big Four”, TRPV1-activating potency is relatively modest for Δ^9^-THC (**1**) and CBC (**4**), but higher for CBG (**3**) and CBD (**2**) [22]. Acidic cannabinoids show modest or negligible affinity for TRPV1, while the shortening of the pentyl chain as in virinoid phytocannabinoids can be associated with increased potency [22,23]. The most potent phytocannabinoid activators of TRV2 are those with the benzochromane skeleton of Δ^9^-THC (**1**), while acidic phytocannabinoids show only marginal, if any, activity. Unlike all other thermo-TRPs, TRPV3 shows sensitization and not desensitization after repeated thermal stimulation, and it is still unclear if chemical activation via ligands is associated with a similar sensitization effect. Δ^9^-THCV (**5a**) and CBD (**2**) show potency similar to carvacrol, the reference activator of TRPV3, while acidic cannabinoids were almost inactive [23]. The viridinic phytocannabinoids Δ^9^-THCV (**5a**) and CBDV (**9a**) showed robust activating properties for TRPV4 but, interestingly, less potent phytocannabinoids (CBG (**3**), CBGV (**10a**), CBGA (**10b**) and CBN (**7**)) showed better desensitizing properties after TRPV4 was activated by 4-αphorbol-12,13-didecanoate (4α-PDD). Many phytocannabinoids also activate TRPA1, in some cases (CBDV, CBC, CBD, Δ^9^-THCA, CBDA and CBG) more potently than the standard activator mustard oil, albeit with minor efficacy, possibly due to only partial agonistic properties [22,23]. Unlike the other thermo-TRPs, TRPM8 is inhibited by phytocannabinoids, with potency especially marked in CBC (**4**), which could completely inactivate the ion channel [22,23]. Given the promiscuous interaction with various thermo-TRPs, especially for CBD (**2**) and THCV (**5a**), it is unclear how this diversity of targets eventually translates in vivo, but the neurobiological significance of TRPV1 activation seems well established for anti-epileptic activity.

#### 2.1.5. Serotonin (5HT) Receptors

THCA (**5b**), THCV (**5a**) and CBDA (**9b**) can modulate the activity of serotoninergic receptors, acting as 5HT3 and 5HT1A allosteric positive modulators [24]. The potentiation of 5HT1A activity has been suggested to underlie the in vivo positive results observed when CBDA was investigated in rodent models of nausea and of Dravet syndrome [25], while the modulation of 5HT3 is important in reducing gastro-intestinal motility and intestinal pain in the animal model of irritable bowel disease [26].

#### 2.1.6. GABA Receptors

γ-Aminobutyric acid type A receptors (GABAARs) are the central nervous system’s (CNS’s) main inhibitory mediators, an activity associated with chloride ion movement [27]. GABAARs are involved in different neurological conditions, and especially anxiety and epilepsy. CBDV (**9a**) shows potent positive modulating activity on GABA-A receptors, possibly secondary to the activation of CB2 and not associated with direct binding [28].

#### 2.1.7. Anti-Inflammatory Targets

The inhibition of the release and/or activity of various pro-inflammatory cytokines and chemokynes (IFN. CSCL8, CDCL10, CCL2, CCL4 and CCL5) and inflammatory mediators was reported for various minor phytocannabinois, often already at the one-digit micromolar concentration [17]. Particularly interesting are the data reported for Δ^9^-THCA-A (**5b**) [29] and CBDA (**9b**) [30]. CBDA inhibited COX-2 with an EC_50_ as low as 2 ¦ĚM and a 9-fold selectivity for COX-2 vs. COX-1. This activity is not surprising, since acidic phytocannabinoids can be viewed as substituted salicylates [31].

#### 2.1.8. PPARs

Dual PPARα/γ activators modulate the lipid metabolism, reducing the accumulation of adipose tissue as well as insulin resistance, and are therefore of relevance for the prevention and management of type-2 diabetes and metabolic syndrome [32]. The acidic phytocannabinoids Δ^9^-THCA-A (**5b**), CBDA (**9b**) and CBGA (**10b**) outperformed their decarboxylated analogues in terms of PPARα/γ modulation potency, showing that a carboxylic group is required for high potency [33]. Δ^9^-THCA-A is the most potent phytocannabinoid PPARα/γ modulator [33,34]. It behaves as a partial and selective PPARγ modulator, showing lower adipogenic activity than full PPARγ agonists such as rosiglitazone [34]. Based on docking and in vitro functional assays, Δ^9^-THCA-A was suggested to bind and activate PPARγ targeting both the canonical and the alternative sites of the ligand-binding domain [34]. Significant PPARγ-modulating activity was also reported for cannabimovone, a phytocannabinoid devoid of affinity for cannabinoid receptors [35], further showing that the structure–activity relationships for interaction with CB1/2 and PPARs are different.

#### 2.1.9. Estrogen Receptors

Via virtual screening, cannabitriol (CBT, **12**) was identified as a powerful ERα antagonist, outperforming tamoxifen [36]. The data should, however, be confirmed in an assay of anti-estrogenic activity.

### 2.2. Phenotypic Assays

#### 2.2.1. Anti-Cancer Activity and Pro-Carcinogenic Activity

Interesting results were reported for CBDA (**9b**). Although less active than CBD when assayed in HL60 (promyelocytic leukemia) and in CEM (promyelocytic leukemia) cells, remarkable activity was observed against MDA-MB-231 cells, a highly aggressive line of triple-negative breast cancer cells [37]. The activity was related to the attenuation of the transcriptional activity of PPARβ/δ, both expressed in MDA-MB-23 cells [38] and of AP-1 [39]. A cannabis extract rich in CBGA showed cytotoxic activity against colon cancer and human leukemia cells. Interestingly, the anti-cancer activity of CBGA (10b) was synergized by both Δ^9^-THC and CBD [40,41]. Overall, the cytotoxic data are rather preliminary, although suggestive of potential discrimination between cancer and normal cells as well as of synergistic activity (*entourage effect*) for phytocannabinoid mixtures.

Low physiological concentrations of CBDV (**9a**) (as well as of CBD) were reported to damage genetic material in human cells, potentially leading to adverse long-term effects such as the induction of cancer and infertility [42]. The results of this study require a careful evaluation to evaluate their clinical implications, especially in the context of a potential Novel Food designation for CBD.

#### 2.2.2. Anti-Bacterial Activity

Phytocannabinoids show potent anti-bacterial activity against Gram-positive pathogens, including those resistant to methicillin [43]. MIC values in the one-digit μg/mL range were reported, along with the strong inhibition of biofilm formation both in Gram-positive and Gram-negative bacteria [44,45,46]. In general, acidic phytocannabinoids were less potent than their neutral analogues, but structure–activity relationships were otherwise somewhat flat, perhaps suggesting membrane-based activity [43,47]. Within the various phytocannabinoids assayed, no compounds significantly outperforming the others emerged, supporting the view of membrane-based activity [43,47]. In keeping with this view, when CBD and CBDV were comparatively evaluated as anti-bacterial agents, no significant difference emerged, except the major sensitivity of *Staphylococcus aureus* to CBD after prolonged (72 h) exposure [45]. CBD showed remarkable helper activity against resistant bacteria, significantly potentiating the effect of bacitracin (BAC) against Gram-positive bacteria, and activity also worth systematically investigating with other phytocannabinoids [4]. Remarkably, CBD did not show any sign of toxicity against mammalian cells when assayed at dosages two orders of magnitude higher than the anti-bacterial ones [48].

### 2.3. Acne

Phytocannabinoids can regulate lipogenesis in sebaceous glands and the proliferation of sebocytes [49]. Thus, THCV inhibited the proliferation of human SZ95 sebocytes with a TRPV4-dependent mechanism [50]. The activation of this ion channel increases intracellular calcium concentrations, stimulating ERK1/2 activity. The reduced production of sebum was associated with a reduction in the intracellular arachidonate levels, providing a strong rationale for clinical studies aimed at the management of acne [50]. The effect of phytocannabinoids on keratinocytes is, however, rather complex, since some of them (CBC, CBG, CBGA and THCV) were found to significantly and differentially affect the expression of various elements of the skin endocannabinoid system [51], the skin endocannabinoid contents, and the activity of enzymes associated with the endocannabinoid system (FAAH (Fatty Acids Amides Hydrolase) and MAGL (Monoacylglycerol Lipase) catalytic activity) [50].

## 3. Preclinical In Vivo Studies

Studies are grouped in this section according to animal models of disease, discussing the rationale for investigating minor phytocannabinoids in these conditions. A summary of the animal data on the pharmacokinetics of some minor phytocannbinoids is also included in this section. No human pharmacokinetics and metabolism data have been reported for “minor phytocannabinoids”.

### 3.1. Epilepsy

Despite the availability of numerous drugs, this condition cannot be satisfactorily managed in ca 1/3 of patients [28]. This, and the development of drug resistance, combine to make the discovery of novel drugs to control seizures a critical urgency. The potential of cannabinoids to be developed as anti-epileptic agents dates to the early studies of Adams on the structure of CBD [1] but was only recently pursued in the wake of the anecdotal beneficial effects of non-narcotic cannabis preparation in various forms of epilepsy [3]. These studies eventually led to the approval of CBD for the management of some juvenile genetic forms of epilepsy by the FDA in USA, the EMA in Europe and medical agencies elsewhere in the world [3]. Additionally, CBDV (**9a**) raised considerable interest as an anti-convulsant agent due to its activity, as a stand-alone agent, in three of the four major rodent models of epilepsy, namely audiogenic-, electroshock-, and pentylentetrazole (PTZ)-induced seizures, failing, however, to show significant activity when pilocarpine was used to induce seizures [28]. On the other hand, activity could be also observed in this model in co-administration studies with valproic acid (VPA) and phenobarbital [28]. No activity was, however, reported in zebrafish models of epilepsy. Activity in the murine models was more marked in age models mimicking preadolescent and the adult state, while potency was lower in models where infancy administration was mimicked. In all studies, the profile of safety was excellent and side effects minimal. The activity was independent from CB1 modulation, and investigation on the expression of epilepsy-related genes showed the upregulation of mRNA coding for a variety of proteins, including the brain-derived neurotrophic factor (BDNF), only in responder animals [28]. The desensitization of TRPV1 by reduced phosphorylation has been suggested to be important for the anti-convulsant activity of CBDV, in line with results observed in TRPV1 knock-out mice, where an attenuated response to CBDV was observed [28]. TRPV1 shows distinct levels of expression with age, and the age-related potency of CBDV as an anti-convulsant agent could be the result of the age-related expression of TRPV1. Despite the lack of in vivo conversion to CBD, CBDA also showed activity in the Scn1aRX/+ mouse model of Dravet syndrome [52]. In these mice, elevated body temperatures induce generalized tonic–clonic seizures that are assumed to recap those observed in children with Dravet syndrome. The intraperitoneal injection of CBDA (10 and 30 mg/kg) caused a significant and dose-dependent increase in the seizure temperature threshold. Protection against electroshock-induced seizures was also reported in rats [53], suggesting that CBDA (**9b**) could be a better anti-epileptic drug than CBD [53]. Potent anti-epileptic activity was also reported for THCV, which could reduce the incidence of seizure induced in rats by PTZ at intraperitoneal dosages as low as 0.25 mg/kg [54].

### 3.2. Autism Spectrum Disorders (ASD)

Based on the observation of a positive association between the use of valproic acid (VPA) during pregnancy and the development of autism in children, the so called “environmental model” of ASD was developed [55]. Thus, prenatal exposure to VPA in pregnant rats causes ASD-like symptoms reminiscent of human ASD (repetitive and stereotypic behaviors, poor learning ability and memory loss). The C3-phytocannabinoid CBDV was investigated in P34 and P58 male offspring of VPA-exposed mothers, and an improvement in ASD-related symptoms was observed (hyperactivity, memory deficit and social behavior) [55]. Interestingly, the early administration of CBDV (**9a**) could completely prevent the appearance of autistic features. In these studies, it was shown that CBDV could restore hippocampal endocannabinoid signaling in the hippocampus, contrasting neuroinflammation and microglia activation [55].

### 3.3. Rett Syndrome (RTTS)

This X-linked dominant disease is the most common cause of female intellectual disability and is associated with the production of a pathogenic variant of MCP2 (methyl CpG Binding Protein 2) [56]. This translates into poor hand skills with stereotypic movements, abnormal gait and poor language skills. Epileptic symptoms often develop to further decrease the quality of life. The hemizygous modification of the involved gene (*PCP2*) could duplicate, in a rodent (mouse) context, the symptoms of the human disease. On account of the anti-epileptic activity of CBDV (**9a**), this compound was investigated in a mouse model of RTTS [56]. The administration of CBDV was associated with a significant improvement in health, associated not only to a decrease in the epileptic crises, but to a general attenuation of the RTTS phenotype, with particularly positive results on memory deficits [56]. The mechanism involved in this beneficial effect remains largely unknown.

### 3.4. Ulcerative Colitis (UC)

The administration of dinitrobenzensulfonic acid (DNBS) induces an intestinal inflammatory status in mice, seemingly mimicking the one observed in UC patients. Oral treatment with CBDV (**9a**) three days after DNBS had a positive effect on histological markers as well as on various biochemical inflammatory parameters (the production of inflammatory cytokines, myeloperoxidase activity and TRPA1 expression) associated with the disease model [57]. Similar results were observed when colitis was induced with dextrane sulfate [57]. Activity was traced to the activation and subsequent desensitization of TRPA1, since the co-administration of a TRPA1 antagonist strongly attenuated the beneficial effects [57]. An expression investigation of this ion channel in colony biopsies from pediatric patients suffering from ulcerative colitis had previously shown an upregulation of TRPA1, rationalizing the detrimental effect of the co-administration of CBDV with the TRPA1 inhibitor HC030031, which prevented the activation and then desensitization of the ion channel by CBDV. CBDV could also affect the microbiota composition, attenuating the dysregulation of gut microbiota associated with colitis [57].

### 3.5. Duchenne Muscular Dystrophy (DMD)

This X-linked recessive disease is associated with a mutation in the dystrophin gene. Its protein product is critical for membrane preservation in muscle fibers, and its deficiency causes chronic inflammation and the irreversible degeneration of skeletal muscles. In a comparative study, intraperitoneal CBD (**2**) and CBDV (**9a**) were investigated in male dystrophic mice, with functional (locomotor tests) as well as biochemical end-points [58]. Both compounds could prevent the impairment or loss of locomotor activity, reducing inflammation and restoring autophagy. The activation of TRPV1 was critical for this activity [58].

### 3.6. Diabetes and Metabolic Syndrome

Interest in this area was triggered by the hypothesis that CB1 partial agonists such as THC could actually behave as functional antagonists in conditions where increased endocannabinoid tone is observed [59]. Thus, 2AG, the major plasmatic endocannabinoid and a full agonist at CB1, is present in high concentrations in diabetic patients presenting visceral obesity. In this condition, THC could actually decrease the endocannabinoid tone and behave as a CB1 antagonist because of its lower efficacy compared to its endogenous version(s) [59]. The association between CB1 inhibition and weight loss was validated with the CB1-reverse agonist rimonabant, but CNS-associated activities make neither THC nor rimonabant clinically suitable as anti-diabetic drugs because of the depressive symptoms associated with their weight-loss activity [59]. Hence, there is interest in THC analogues with a different profile of affinity for CB1. In rodent studies, THCV (**5a**) induced hypophagia and weight reduction in lean mice, improving insulin sensitivity and glucose tolerance in obese mice [60]. Increased insulin sensitivity was also observed in genetically (ob/ob) obese mice, without, however, weight loss [60]. Additionally, Δ^9^-THCA-A (**5b**) was investigated as an anti-diabetic agent, improving glucose tolerance and attenuating liver fibrosis, the hallmark of non-alcoholic fatty liver disease (NAFLD), in HFD mice [34]. Fibrosis was also significantly attenuated in the CCl_4_-induced liver damage model [61]. A reduction in body weight and adiposity and in immune cell infiltration was also observed, along with a significant browning effect on white adipose tissue [34,61]. In accordance with the reduced affinity of acidic phytocannabinoids for thermo-TRPs compared to their neutral analogues, activity was associated with interaction with PPARγ and not to TRPV1 [61].

### 3.7. Nausea

CBDA (**9b**) could inhibit nausea and vomiting elicited in rats by various toxins and by movement, outperforming CBD by three orders of magnitude [24,62]. The activity was traced to the activation of 5-HT1A receptors. In a separate study, CBDA was found to potentiate the anti-emetic activity of metoclopramide in a lithium-chloride-induced conditioned gaping nausea model in rats [63]. Similar results were obtained with CBDV (**9a**) (200 mg/Kg) and THCV (**5a**) (20 mg/Kg) [63].

### 3.8. Analgesic and Anti-Inflammatory Activity

Many minor phytocannabinoids show potent analgesic and anti-inflammatory activity. Δ^9^-THCA-A (**5b**) was active in a rodent model of arthritis in a CB1- and PPAR-γ activating mode [61]. CBN could attenuate the production of inflammatory interleukins (IL-2, 4, 5 and 13) and decrease the production of allergen mucus in ovalbumin-sensitized A/J mice [64,65]. Δ^9^-THCA-A showed potent anti-inflammatory activity in mice fed a high-fat diet (HFD), reducing the expression of the inflammatory agent tumor necrosis factor alpha (TNF-α4) and of the cytokine interleukin 10 (IL-10), an activity related to PPARγ stimulation [61]. Δ^9^-THCV administered intraperitoneally at a dosage of 1 mg/kg could attenuate the signs of inflammation and hyperalgesia induced in mouse hind paws by the intraplantar injection of carrageenan or formalin [66]. The administration of CB1 or CB2 receptor antagonists (SR141716A and SR144528, respectively) had a detrimental effect on these activities, suggesting the involvement of both GPCRs in these activities [66]. CBN showed a beneficial effect in chronic muscle pain conditions such as temporomandibular disorders and fibromyalgia in a rodent model of myofascial pain (nerve growth factor injection in the masseter muscle) [64]. Finally, CBDA administered intraperitoneally at a dosage of 10 mg/kg could prevent the carrageenan-induced inflammatory response in rodents, also decreasing carrageenan-induced hyperalgesia [67]. Taken together, these studies show that pain and inflammation are a major, and still unexploited, target for “minor phytocannabinoids”.

### 3.9. Cachexia

CBN (7) induced hyperphagia and increased food consumption and feeding time in rats [68] The activity was lower than that of Δ^9^-THC, but, since CBN is not narcotic, CBN could have better potential as an orexic agent in conditions where appetite stimulation is beneficial, such as in terminal cancer and HIV patients.

### 3.10. Neuroprotective Activity

THCA (**5b**) was active in a murine model of Huntington’s disease. Again, activity was associated with the activation of CB1 and PPAR-γ [69]. In a rat Parkinson model, THCV could significantly reduce motor movements induced by 6-hydroxydopamine [70].

### 3.11. Glaucoma

Cannabicitran (11, Figure 2) was reported to reduce intraocular pressure in rabbits [71]. Cannabicitran does not interact with cannabinoid receptors [17], and the activity was associated with the activation of GPR18 (NAGly receptor), an important target for glaucoma.

### 3.12. Sleep Induction

CBN (7) is the main degradation product of Δ^9^-THC, and high concentrations of CBN are found in aged cannabis products. For unclear reasons, CBN has been associated with sleep induction, and CBN was marketed, at dosages generally lower than 5 mg/die, as “the sleepy cannabinoid in old weed” [72]. However, sleep studies on CBN did not come to a clear conclusion regarding this claim. In one study, CBN was reported to increase barbiturate-induced sleep time [73], but the observation could not be replicated in a following study [74]. However, when CBN was administered in combination with Δ^9^-THC, a synergistic increase in sedation was observed in a murine study [75], which could be replicated in a small clinical trial (See Section 4.7).

### 3.13. Tetrad Test

This combination of four rodent behavioral assays (the induction of catalepsis and hypothermia, hypolocomotive and anti-nociceptive activity) is a strong indication of mostly CB1-related cannabinomimetic activity. THCV (**5a**) [76] as well as (−)-Δ^9^-*cis*-THC (**6**) [16] proved active in this assay, suggesting residual CB1-activating properties for THCV. Modest activity could also be observed with CBDA, probably associated with 5HT1A enhanced activation [25].

### 3.14. Pharmacokinetic Studies

Absorption and metabolism data are only available for a few “minor phytocannabinoids”. The native acidic version of the “Big Four” (THCA, CBDA, CBGA and CBCA) and of two of their varinoid versions (CBDVA and CBGVA) were investigated following intraperitoneal administration in mice, evaluating their brain and plasma profile [52] An oily formulation was used, and absorption was, in general, rapid (t_max_ = 15–45 min), with a short half-life (<4 h) and minimal brain penetration (brain–plasma ratios < 0.04). However, formulation in a tween-based vehicle dramatically increased the brain penetration of CBDA [52]. Remarkably, no absorption of THCA could be observed when the compound was formulated in a tween-based vehicle. This ruled out the concept that the increased brain penetration of CBDA was due to encapsulation in tight-junction-permeable micellar-like structures, since a similar effect should have also been observed in THCA. A better human absorption of CBDA vs. CBD has also been claimed in the patent literature [77].

CBD strongly inhibits several classes of CYP450 isoenzymes, and the varinoids CBDV and THCV were investigated for the inhibition of the four most common isoforms of CYP2D6. This cytochrome is responsible for the oxidative metabolization of the endocannabinoid anandamide as well as several anti-depressants and anti-psychotic drugs and is expressed both in the liver and in the brain. The binding of the two compounds differed significantly between the four variants but was, in any case, lower than the one of Δ^9^-THC [78].

## 4. Clinical Studies

The preclinical profile of some minor phytocannabinoids qualifies them as potential priority candidates for several conditions whose clinical management is currently unsatisfactory in terms of efficacy and/or tolerability. However, definite clinical evidence of activity is currently largely missing, since the positive studies reported so far are too small and/or focused on proxy pathological end-points, while some of the studies never met their end-point(s). Within minor phytocannabinoids, clinical data are only available for divarinyl phytocannabinoids (C3-phytocannabinoids) and for CBN. As is discussed in Section 5.1, by selective breeding, it was possible to generate cannabis varieties which accumulate large amounts of a specific C3-phytocannabinoid, sufficient to sustain clinical studies and to provide a viable alternative to total synthesis. Clinical investigations have so far been carried out for CBDV and Δ^9^-THCV (5a). The receptor profile of CBD and CBDV are qualitatively similar, if not overlapping, (see Section 2.1), and both compounds show poor oral absorption (5–10%), extensive protein binding and allylic oxidation at C-6 and C-7 as part of their metabolism [78]. On the other hand, the human metabolism of CBDV (**9a**) is poorly characterized, while the role of metabolites in the clinical activity of CBD has not yet been definitely settled. The possibility therefore exists that differences related to metabolization and/or to different relative potency could translate into a better clinical profile for CBDV. These observations provided a rationale for the clinical investigation of CBDV in disabling neurological conditions where beneficial effects for CBD have been clinically demonstrated or suggested from preclinical studies (see Section 3.1). Since no CBD-CBDV comparative studies are available, it is difficult to evaluate if, and to what extent, CBDV could have clinically outperformed CBD in the reported clinical studies. On the other hand, the preclinical profiles of Δ^9^-THC and Δ^9^-THCV are non-overlapping [76], providing a solid rationale for investigating Δ^9^-THCV in diseased states not covered by the profile of its parent major C5-phytocannabinoid Δ^9^-THC.

### 4.1. Autism Spectrum Disorders (ASD)

This disorder is ill-defined in terms of molecular and mechanistic bases, but there seems to be a general consensus that a dysregulation of the brain excitatory and inhibitory systems triggers its insurgence and maintains its associated phenotypic profile. In turn, this dysregulation mirrors, or is associated to, a dysregulation of the endocannabinoid system. The activity of CBDV (**9a**) to modulate the brain excitatory–inhibitory system was first investigated in a double-blind, randomized, placebo-controlled crossover clinical study whose results were reported in 2019 [79]. The quantification of the brain concentration of glutamate, the major excitatory amino acids, GABA, the major inhibitory amino acid and their metabolites served as the end-point of a double-blind randomized study that involved 34 volunteers, of which 17 were men with autism spectrum disorder (ASD) and an equal number were adults who did not have autism. Participants were orally administered a placebo or a 600 mg dosage of CBDV daily. Measurements were carried out via magnetic resonance in the brain area allegedly involved to the phenotypical development of autism (dorsomedial prefrontal cortex and left basal ganglia) starting 2 h after the treatment, when the peak concentration of CBDV was reached, and with at least 13 days between sessions to secure wash-out for the crossover part of the study. CBDV significantly increased the glutamate concentration in the basal ganglia in both arms of the study, while the effect was negligible in the dorsomedial prefrontal cortex, and no change was observed for the GABA levels. The increased glutamate concentration correlated negatively with the baseline concentration of this metabolite, being higher when the baseline concentration was low, only in the autism branch, but not in the control arm of the study. Overall, the results of this first study showed that CBDV could selectively modulate the glutamate concentration in the basal ganglia and modulate the excitatory–inhibitory balance of brain systems both in non-autistic and in autistic people, with a negative correlation between the increase and the basal levels selectively observed only in the autistic branch of the study [79].

A second clinical study was reported by the same group in 2021, focusing on the magnetic resonance assessment of the striatum (caudate, nucleus accumbens and putamen) functional connectivity, which is apparently atypical in autistic patients [80]. To this purpose, 28 volunteers were recruited, 13 who had autism and the remaining serving as the control in a double-blind, placebo-controlled, crossover study. The same CBDV dosage (600 mg die) of the previous study was used, also with the same wash-out period associated with crossover. Compared to the neurotypical volunteers, those with autism had an overall lower functional connectivity between the ventral striatum and the frontal and pericentral brain regions. These are associated with emotion and motor and vision processing). Intra-striatal functional connectivity was higher, as was the one of the putamen with the temporal areas responsible for speech and language. The administration of CBDV modulated the atypical striatal circuitries observed in autistic patients towards a more neurotypical profile [80]. Taken together, the results of these two studies show that CBDV can selectively modulate brain activity in autistic patients. Additional studies will, however, be necessary to evaluate if, and how, the normalization of the glutamate to GABA ratio and of striatum functional connectivity translates in terms of a symptomatic improvement of autism symptoms.

### 4.2. Epileptic Conditions

Interest in the anti-epileptic activity of minor cannabinoids was fueled by a case study reported in 2016 on a patient with drug-resistant epilepsy who was experiencing a significant improvement in his conditions by self-administrating cannabis in combination with mainstream therapy. The withdrawal of cannabis significantly worsened the state of the disease, and, although the exact phytochemical profile of the cannabis preparation(s) used was unclear, HPLC analysis of the plasma cannabinoid profile evidenced a high concentration of CBDV (**9a**). Although CBDV could simply be a marker of cannabis consumption by the patient, this, coupled with preclinical evidence, spurred controlled studies on the anti-epileptic activity of CBDV [81]. A major double-blind, randomized, and placebo-controlled study was carried out on 162 adult patients with poorly controlled seizures. The protocol involved a baseline period of four weeks, participants next titrated from 400 to 800 mg CBDV (or placebo) twice daily (b.i.d.) over two weeks. Next, a six-week period of constant dosing (800 mg b.i.d.) followed, and a twelve-day taper period concluded the study. CBDV showed a satisfactory profile of safety and side effects, but the primary endpoint of the study, namely a different frequency of focal seizures in the treatment and the placebo arms of the study, was not met, due to a surprisingly high placebo response [82]. Although the extent of seizure reduction observed (40.5%) was remarkable, its statistical significance was marred by a surprisingly high placebo response. The aura of a “miracle drug” surrounding cannabis and the high expectations of patients for cannabis-based medication could have contributed to the, overall, negative results of the study. On the other hand, highly positive results, better than those observed with CBD, have been claimed in the patent literature for CBDA, in accordance with preclinical observations [77]. Additionally, worth mentioning are the preliminary positive results in terms of safety and efficacy observed when five patients with Rett syndrome, a condition often associated with drug-resistant epilepsy crises, were treated daily with 10 mg/kg CBDV [83].

### 4.3. HIV-Associated Neuropathic Pain

Despite the success of anti-retroviral therapy to decrease HIV mortality, the quality of life of patents is often far from satisfactory, with neuropathic pain remaining the major, and clinically largely unmet, neurological complication of the infection. The efficacy of CBDV (9a) to manage HIV-associated neuropathic pain was investigated in a randomized, placebo-controlled, double-blind and crossover study in 32 HIV patients with HIV-associated neuropathic pain [84]. The study involved two distinct treatment phases, each lasting four weeks, with a dosage of 400 mg of CBDV (or placebo) die and a three-week washout period to prevent carry-over effects. Pain intensity, the primary end-point, was measured on an eleven-point numeric scale, following patients for three weeks after their second phase of treatment. The need to additional pain medication, the pain features and the overall quality of life were considered as secondary end-points. Surprisingly, the treatment with CBDV was associated with a higher mean pain intensity compared with the placebo, while no difference was observed in the secondary end-points. No adverse reactions were associated with treatment with CBDV, in line with a similar safety profile observed in the other clinical studies on this compound [84]. The results of this study confirm that significant analgesic activity in phytocannabinoids is associated with interaction with CB1, not a recognition site for CBDV [17].

### 4.4. Obesity

Obesity is the result of the excessive storage of fat in adipose tissue, a condition associated with a low-grade chronic inflammatory status. There is anecdotal evidence that cannabis could be beneficial, at least from a long-term perspective, for the management of obesity [59,85] Thus, chronic users of marijuana seem to have a paradoxical lower body mass index compared to non-users, despite the stimulation of appetite associated with cannabis smoking, and several reasons have been put forward, but not yet proved, to back up this surprising correlation [85]. On the other hand, the observation that the CB1 reverse agonist rimonabant shows solid clinical evidence of anti-obesity activity triggered interest in Δ^9^-THCV (**5a**), since, as a seemingly neutral CB1 receptor antagonist, it should not negatively affect neural reward responses and induce depression [85]. To test this idea, prematurely exploited in the cannabis marked by dubbing THCV as the “weed diet”, a small within-subject but double-blinded study was initially carried out on twenty healthy volunteers who received a single oral dose of 10 mg Δ^9^-THCV or placebo on two different occasions. The end-point of the study was the comparative neural response to a sensory reward (the sight and/or flavor of chocolate) and to a sensory aversive stimulus (a moldy strawberry picture and/or an unpleasant strawberry taste) as assessed using functional magnetic resonance imaging. The volunteers were also asked to rate the pleasantness, intensity and desire for each stimulus. Δ^9^-THCV could increase the response to the positive stimulus in several brain areas (mid-brain, anterior cingulate cortex, caudate and putamen) and to the negative one in the amygdala, insula, mid orbitofrontal cortex, caudate and putamen. These observations suggest that Δ^9^-THCV can modulate the neural reward pathways without the induction of the psychoactive effects associated with Δ^9^-THC [86].

In a second study, the capacity of Δ^9^-THCV to normalize neural functional connectives altered in obese patients was investigated via magnetic resonance imaging, focusing on the amygdala, the insula, the orbitofrontal cortex and the dorsal mid-prefrontal cortex, which are areas involved in the reward pathways. The study was small (twenty healthy volunteers) but randomized, within-subject and double-blind, and it also involved the evaluation of mood and subjective experience using self-reported scales [87]. The oral administration of a single 10 mg dose of Δ^9^-THCV was not significantly different from the placebo in terms of subjective experience, as expected for a non-psychoactive compound. Nevertheless, a reduction in resting state functional connectivity was observed between the amygdala and the dorsal anterior cingulate cortex and between the dorsal mid-prefrontal cortex and the frontal and medial gyrus. Remarkably, functional connectivity is altered in an opposite way in these areas, suggesting a potential for the management of obesity.

### 4.5. Diabetes

THCV (**5a**) and CBD (**2**) were comparatively and associatively investigated in a small clinical study in dislipidemic type 2 diabetes patients [88]. The study was randomized, double-blind and placebo controlled and involved 62 volunteers not under insulin treatment. Patients were sorted into five arms, treated for 13 weeks with CBD (2 × 100 mg/die), THCV (2 × 5 mg/die), a 1:1 combination of CBD and THCV (2 × 5 mg each die), a 20:1 combination of CBD and THCV (2 × 100 mg CBD + 5 mg THCV daily) and a matched placebo. The primary end-point was an increase in HDL-cholesterol (HDL-C) from the baseline value, with a constellation of secondary and tertiary end-points (glycemic control, lipid profile, insulin sensitivity, body weight, liver triglycerides, appetite, inflammation and vascular function markers, distribution of adipose tissue, gut hormones and the plasma level of endocannabinoids and adipokine). Overall, the complete set of end-points was not met in any of the branches, but significant improvement in some of the secondary and tertiary end-points was associated with the cohorts receiving THCV as a stand-alone treatment (fasting glucose level, glucose response, HOMA β-cells function, plasma levels of adiponectin and Apo-A). On the other hand, no significant deviation from the placebo levels was observed in the CBD branch, although some biochemical parameters were improved compared to the baseline level. Significant side-effects were not observed in any branch of the study [88]. Taken together, the results of this study show that THCV warrants further studies as an anti-diabetic agent, but important issues still remain unresolved. Thus, a raised concentration of RBP-4, an adipokine associated with obesity and insulin resistance, was paradoxically observed in the THCV branch of the study. This activity was apparently offset by the raised concentration of adiponectin, an adipokine that increases insulin sensitivity and whose production is decreased in obesity and diabetes. More difficult to explain is the significant increase in Apo A, but not in HDL-cholesterol, since Apo A represents up to 90% of the protein composition of HLD-C. On the other hand, in sharp contrast with the CB1 reverse agonist rimonabant, no significant effect was observed on body weight, suggesting a distinct mechanism of modulation of the endocannabinoid system. Unlike rimonabant, THCV can also indirectly modulate the tone of the endocannabinoid system [17], decreasing it in a direct fashion when high concentrations of 2AG are present, but also indirectly increasing its tone by inhibiting the degradation of the endocannabinoids by FAAH and MAGL. The results observed in the study do not seem directly related to a modulation of the ECS. Finally, also puzzling was the observation that the beneficial effects of THCV were lost when THCV was associated with CBD, a counterintuitive observation in the light of the still largely unproved and possibly inflated claim that phytocannabinoids show synergistic activity [88].

### 4.6. Addiction

As a seemingly neutral CB1 antagonist, THCV (**5a**) could, in principle, reverse the neurological side effects associated with THC intoxication, and the idea was tested in a small study, which, however, provided contrasting results. The study was placebo-controlled, double-blind and a crossover and involved ten male cannabis users, dosed with THCV (10 mg) for five days, and then with 1 mg of intravenous Δ^9^-THC on the fifth day [89]. The end-point was a reduction in the intoxicating symptoms induced by cannabis consumption. Compared to the placebo, THCV could reduce the subjective effects of Δ^9^-THC intoxication in nine out of the ten volunteers. Some objective responses were also dampened (delayed verbal recall and increased heart rate), but memory intrusions, that is, memory retrievals that are not merely unintentional, but that are counter-intentional, were, surprisingly, significantly increased. These data show that THCV can moderate some effects of Δ^9^-THC, but also potentiate others [89], and further, larger studies will be necessary to evaluate the possibility to treat addiction to cannabis, a growing issue of public health, with THCV.

### 4.7. Sleep Induction

In one small clinical study involving a small number of participants, CBN (7) potentiated the sedative activity of Δ^9^-THC. The study involved five male volunteers who were dosed with oral CBN (50 mg), Δ^9^-THC (12.5 mg), or two different combinations of the two (12.5 mg Δ^9^-THC + 25 mg CBN or 25 mg Δ^9^-THC and 50 mg CBN), with a wash-out period of one week between the various administrations [75]. The combination did not alter the effects of Δ^9^-THC on heart, blood pressure and body temperature, as well as pain threshold and skin sensitivity, which were not affected by CBN alone [75]. On the other hand, the combination caused a modest increase in some subjective end-points (drowsiness and dizziness), much less marked than those evidenced in a previous animal study (see Section 3.12). The sleep-inducing properties of CBN remain therefore unsubstantiated in terms of clinical validation [72,90].

## 5. Strategy to Increase the Availability of Minor Phytocannabinoids

Within “minor phytocannabinoids”, practically only acidic phytocannabinoids can be obtained in high yield by isolation, being the major native constituents of the cannabinome. The isolation of all the other “minor phytocannabinoids” is complicated by their occurrence in trace amounts and by chromatographic behavior very close to that of the “Big Four”. As a result, direct isolation from landrace cannabis is low-yielding and time-consuming, and several strategies have been developed to increase the availability of minor phytocannabinoids.

### 5.1. Selective Breeding and Metabolic Engineering of Cannabis

By selective breeding, it has been possible to obtain cannabis strains accumulating the viridinoid version (C3-phytocannabinoids) of the “Big Two” (Δ^9^-THC and CBD). The breeding experiments have capitalized on Far-East native strains of the plant, which are richer in viridinoids compared to the European strains, and have resulted in the generation of strains which accumulate a single major phytocannabinoid, greatly simplifying the purification process [91]. The development of strains accumulating the *O*-methylated versions of the “Big Four” could probably be possible by breeding, or, alternatively, in engineered plants overexpressing *O*-methylating enzymes. It is, however, unclear if this strategy can also be applied to other types of minor phytocannabinoids, since the selective overexpression of specific oxidases will be required. On the other hand, since cannabis is a dioecious plant and is anemophilous, it will be difficult to isolate mutants from chemical mutagenesis [92]. In addition, most hemp varieties do not self-pollinate, complicating the obtainment of homozygous plants by conventional self-pollination. Breeding could, however, be dramatically accelerated by genome editing. Thus, by the CRISPR-mediated selective mutation of a specific gene in both alleles, homozygous plants could be obtained in a single editing step, and the future metabolic engineering of hemp could therefore significantly contribute to the availability of minor phytocannabinoids by isolation.

### 5.2. Synthetic Biology

The cannabinoid-building enzymatic and regulatory protein complex has been expressed in yeasts, and, by changing the acyl starter, phytocannabinoids with both even and odd alkyl substituents could be obtained [93]. The “Big Four” are the precursors of minor phytocannabinoids, and by expressing additional enzymes, other classes of compounds could be obtained. For instance, the expression of a methyl transferase could afford mono-*O*-methyl phytocannabinoids, such as *O*-methyl-Δ^9^-THC (13). Of special interest would be the expression of an allylic hydroxylase selective for the nuclear allylic methyl of CBD and THC, since these compounds are major metabolites of the native phytocannabinoids, and their synthetic obtainment is still underdeveloped in terms of overall yield and the number of steps required [94].

### 5.3. Semi-Synthesis

Starting from the “Big Four”, a variety of minor phytocannabinoids can be obtained, mostly via oxidative reactions, UV irradiation or acidic treatment. Conditions for the selective formation of dominant, or exclusive, reaction products have been developed for some “minor phytocannabinoids” [95]. Others, such as iso-THC, can only be obtained in mixture with related compounds, and additional activities will be necessary to improve their availability.

#### 5.3.1. O-Methylphytocannabinoids 

**Scheme 1:**
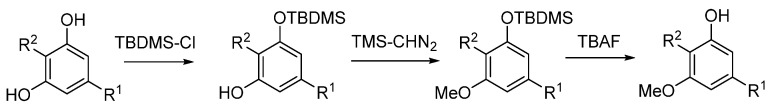
Synthesis of *O*-methylphytocannabinoids. R^1^ = *n*-pentyl; R^2^ = 2,8-menthadien-3-yl (CBD-OMe) or geranyl (CBG-OMe).

Very little information is available on these phytocannabinoids, difficult to obtain by isolation. To increase their availability, a semi-synthesis was developed. In principle, CBD and CBG-type phytocannabinoids could be converted into their corresponding mono-*O*-methyl derivatives via deprotonation and methylation. However, mixtures of natural *O*-methyl and unnatural dimethyl derivatives are obtained. The dimethyl derivatives can be easily removed via chromatography, but an alternative strategy involves the selective protection of one of the two phenolic hydroxyls with a bulkyl silylating agent followed by treatment with trimethylsilyldiazomethane and desilylation was developed [96]. Due to the steric hindrance of the silylating reagent, only monosylylation was observed, and the overall yield was in the range of 40–60% (Scheme 1).

#### 5.3.2. Cannabinol (CBN, **7**)

**Scheme 2:**
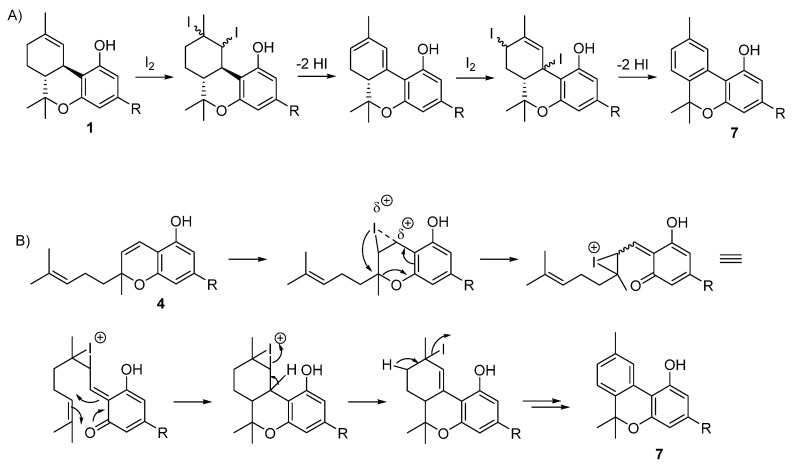
(**A**) Iodine-promoted cyclization of Δ^9^-THC (**1**) to CBN (**7**); (**B**) iodine-promoted prenylchromene-benzochromane rearrangement of CBC (**4**) to CBN (**7**). R = n-pentyl. The two terminal arrows indicate additional dehydrogenation, for which 1,4-addition of iodine followed by two-fold dehydrohalogenation seems plausible [97].

The treatment of Δ^9^-THC (**1**) with iodine directly affords CBN (**7**) as a result of a series of iodination–dehydroiodination steps whose driving force is the eventual menthyl to *p*-cymyl aromatization [97]. The reaction can also be carried out starting from CBD or CBC, with overall yield in the range of 50–70%. With the former, in situ cyclization to THC takes place due to the acidic medium, while with CBC, the addition of iodine to the chromene bond triggers the electrocyclic opening of the heterocyclic ring followed by a hetero Diels–Alder reaction to generate a tetrahydrocannabinol derivative, next aromatized by a set of iodine addition–iodidric acid elimination reactions (Scheme 2) [98]. This chemistry was deftly exploited to develop a one-pot total synthesis of CBN from citral and olivetol in overall ca 70% yield (see Section 5.4) [98].

#### 5.3.3. Cannabicyclol 

**Scheme 3:**
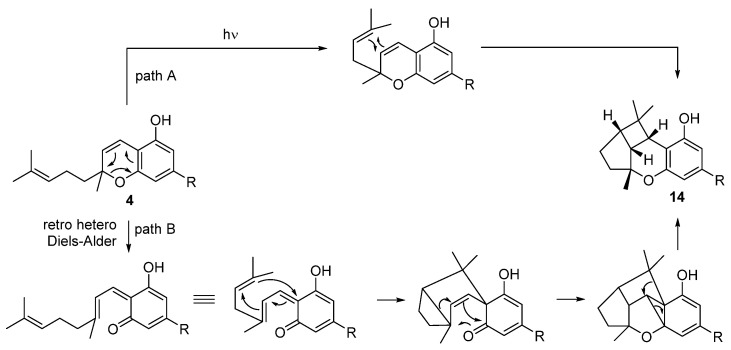
Cyclization of CBC (**4**) to CBL (**14**) under photochemical (path A) and acidic (path B) conditions. R = *n*-pentyl.

The treatment of CBC (**4**) under both acidic and photochemical conditions affords cannabicyclol (CBL, **14**, Figure 2)in ca. 30% yield (Scheme 3). The reaction is a mainstream [2πs + 2πs] cyclization under photochemical conditions but a more complex reaction under acidic treatment, which triggers the retro-electrocyclizative opening of the chromene ring, followed by a thermally allowed [4πs + 2πs] cycloaddition. A homo-1,5-sigmatropic rearrangement then generates a cyclopropane ring, whose cleavage regenerates the aromaticity of the benzene ring [98].

#### 5.3.4. Cannabicitran (CBT, **11**)

**Scheme 4:**
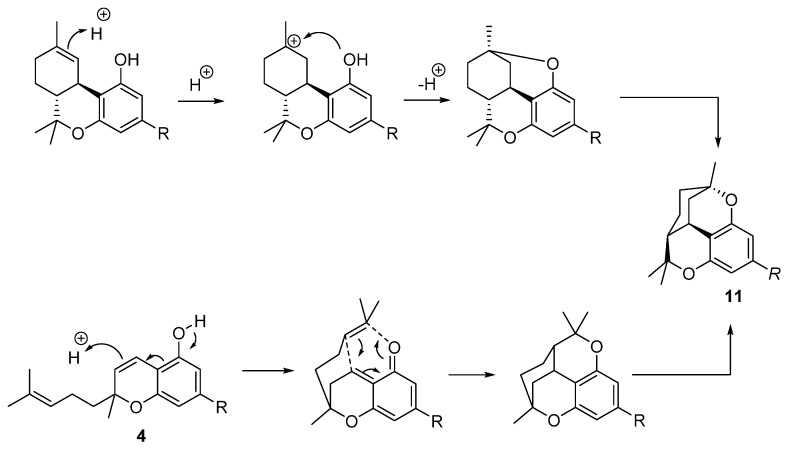
Acid catalyzed cyclization of of Δ^9^-THC (**1**) and CBC (**4**) to CBT (**11**). R = *n*-pentyl.

This compound can be viewed as the result of an additional intramolecular cyclization of both Δ^9^-THC (**1**) and CBC (**4**) [99], and can, indeed, be obtained from both compounds, albeit in a modest (<10%) yield. [95]. While natural CBC is racemic or highly scalemic [99], natural Δ^9^-THC is enantiomerically pure [100]. Cannabicitran by isolation is racemic, suggesting therefore an in vivo derivation from CBC rather than from Δ^9^-THC (Scheme 4).

#### 5.3.5. Iso-THC (**15**)

**Scheme 5:**
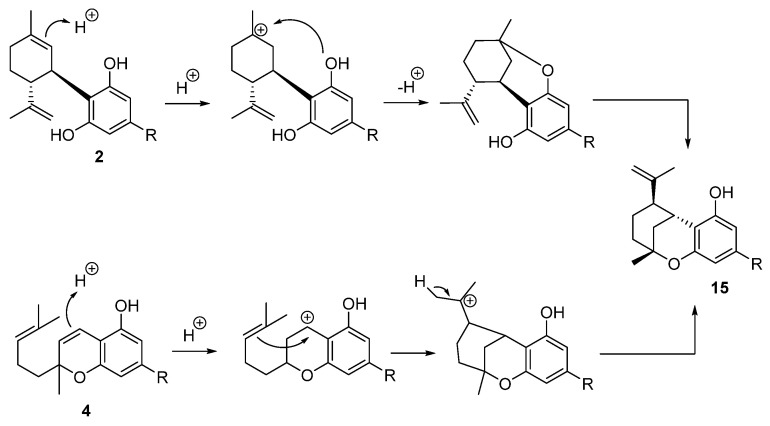
Acid-catalyzed cyclization of CBD (**2**) and CBC (**4**) to iso-THC (**15**). R = *n*-pentyl.

This non-euphoric isomeric form of the narcotic principle of marijuana can be obtained by the acidic treatment of CBD (**2**), or, alternatively, CBC (**4**), although yields are poor (<5%) [101]. The protonation of the endocyclic bond of the menthyl moiety of CBD generates a tertiary carbocation, next trapped by the phenolic hydroxyl of the aromatic moiety. Alternatively, the protonation of the chromene double bond of CBC affords a benzyl cation stabilized by a quinonmethide resonance form, next attacked in a fashion by the isoprenyl terminal bond (Scheme 5).

#### 5.3.6. Furanoid Phytocannabinoids and Cannabimovone (**16**)

**Scheme 6:**
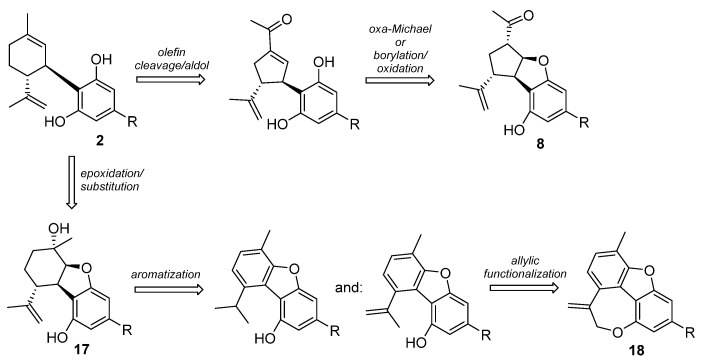
Semi-synthesis of the furanoid phytocannabinoids anhydrocannabimovone (**8**), cannabielsoin (**17**) and cannabioxepane (**18**). R = *n*-pentyl.

Cannabielsoin (**17**, Figure 2), a hemp constituent as well as a mammalian metabolite of CBD [102], and anhydrocannabimovone (**8**) [103] are the best-known phytocannabinoids in which the 1-hydroxyl of the resorcinyl moiety is cyclized on the terpenyl moiety. They are both derived from cannabidiol (Scheme 6) by epoxidation and intramolecular 5-*exo* cyclization (cannabielsoin, **17**) [102] or, after the oxidative cleavage of the *p*-menthyl moiety, by the aldolization of the resulting keto-aldehyde to cannabimovone (**16**, Figure 2). Cannabimovone is unstable and is easily crotonized to an enone, eventually trapped in a Michael fashion by the phenolic hydroxyl to generated anhydrocannabimovone [103] (Scheme 6). Cannabioxepane (CBX, **18**) [104] can be obtained by cannabielsoin by aromatization followed by formal anti-Markovnikov addition to the exocyclic double bond, presumably mediated by epoxidation and a SN2-type opening to overcome regiochemistry issues [105]. The multistep synthesis of furanoid phytocannabinoids and cannabimovone is only efficient for cannabielsoin (overall yield ca 50%) [105], while overall yields <10% have been reported for all other compounds of the class [105].

### 5.4. Total Synthesis

**Scheme 7:**
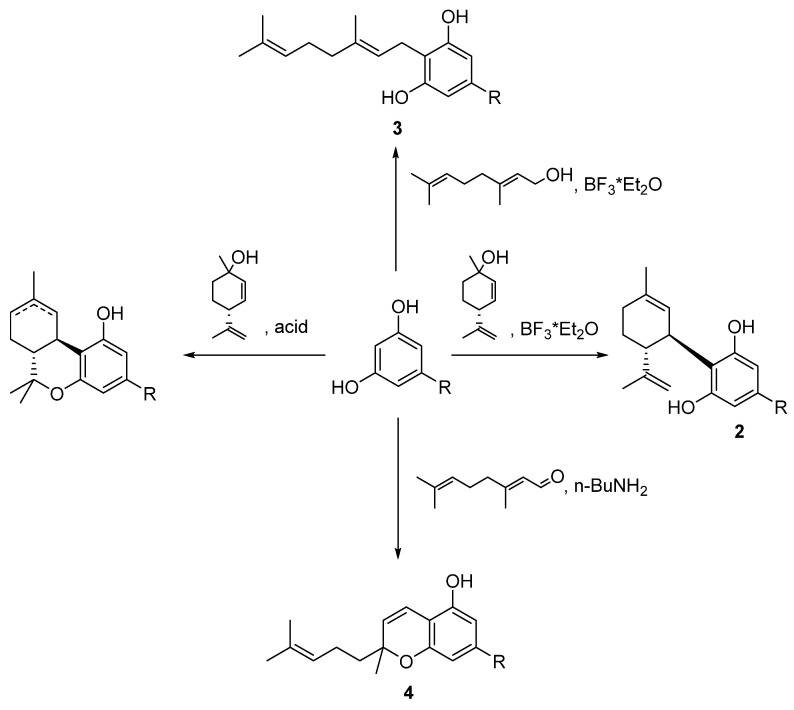
Generation of the diversity of phytocannabinoids by isoprenylation of alkylresorcinols. R = *n*-pentyl.

The “Big Four” can be obtained by straightforward total synthesis, with the length of the alkyl group set by the starting alkylresorcinol (olivetol (C5-phytocannabinoids), orcinol (C1-phytocannabinoids), divarinol (C3-phytocannabinoids), 5-butylresorcinol (C4-phytocannabinoids), sphaerophorol, C6-phytocannabinoids), some of which are commercially available [95,106]. Alkylresorcinols are next isoprenylated with a geranyl donor (geraniol, geranyl bromide) to obtain compounds related to CBG, with a menthadienyl donor (*p*-mentha-2,8-dien-1-ol) to obtain compounds related to CBD and THC and with citral to obtain compounds correlated to CBC. The overall isoprenylation yields are highest for CBC and related compounds (ca 70%) and lower, but still acceptable, for compounds related to CBG (40–60% depending on the acid used), and to CBD and Δ^9^-THC (ca 40–50% yield) [95,106]. The acidic treatment of compounds of the menthyl-type next affords compounds related to Δ^8^ or Δ^9^-THC depending on the reaction conditions. Starting from the synthetic “Big Four”, the conversion into minor phytocannabinoids can be carried out according to various strategies summarized in Scheme 7 [10,95,106].

## 6. Conclusions

The preclinical profile of some minor phytocannabinoids is interesting and predictive of a potential to address complex neurological and metabolic conditions such as diabetes, obesity and autism. Promising preliminary results have been obtained in small clinical studies for all these conditions, and additional activities are also surely warranted in the realm of epilepsy management. However, the small size of these studies, the use of proxy end-points and the observation of a phenotype non-completely overlapping the one predicted by in vitro and the preclinical data combine to suggest a complex pharmacology, only partially recapped in the preclinical investigations and in receptor assays, and needing confirmation in large clinical studies. These issues are very important, since in the metabolism/obesity and neurodegenerative areas it is not uncommon that preclinically promising compounds turn out to be devoid of real clinical relevance. Compensation by other pathways, differences between human diseases and their animal models and unanticipated side effects are major causes of failure [107].

Even with these limitations, THCA-A, THCV and CBDV are definitely promising “minor phytocannbinoids” in terms of clinical potential. THCA-A, like all acidic phytocannabinoids, is non-narcotic and does not generate Δ^9^-THC after oral administration. Nevertheless, due to its easy decarboxylation during storage, it does not qualify for development. Efforts should therefore be directed at finding stable analogues that duplicate the biological profile of acidic phytocannabinoids but cannot undergo decarboxylation. In general, scarce information is available on the pharmacokinetic of minor phytocannabinoids, and this gap should be filled, since absorption and metabolization could substantially deviate from the one associated with the “Big Four”. Furthermore, orally administered phytocannabinoids have low absorption, and therefore, a significant colonic concentration can build up, with interaction with the microbiome providing additional interesting clues to rationalize their bioactivity. Finally, owing to the low concentration of “minor phytocannabinoids” in recreational cannabis, assumptions of safety associated with long-term use cannot be made for these compounds, and the lack of toxicological data is, indeed, one of the major gaps into our knowledge of these compounds. This lack is worrisome, since many of the more interesting potential clinical uses of “minor phytocannabinoids” are in the management of chronic conditions, for which prolonged treatments are necessary. On the other hand, strategies to increase the availability of minor phytocannabinoids have been developed, and many of them are now available to the biomedical community for the additional studies necessary to secure their safety and clinically validate the promising indications from the animal studies.

## Data Availability

Not applicable.
